# Dexmedetomidine alleviates intestinal ischemia/reperfusion injury by modulating intestinal neuron autophagy and mitochondrial homeostasis via Nupr1 regulation

**DOI:** 10.1186/s10020-024-00952-2

**Published:** 2024-11-06

**Authors:** Qiong Wu, Qiuhong Chen, Sisi Liang, Jinping Nie, Yingjie Wang, Chenlu Fan, Zhen Liu, Xuekang Zhang

**Affiliations:** Department of Surgery and Anesthesia, The First Affiliated Hospital, Jiangxi Medical College, Nanchang University, No. 17, Yongwai Zhengjie, Donghu District, Nanchang, Jiangxi 330006 China

**Keywords:** Intestinal ischemia/reperfusion injury, Dexmedetomidine, Nupr1, Autophagy, Mitochondrial homeostasis, Neuron

## Abstract

Intestinal ischemia/reperfusion injury (I/R) is a common yet challenging-to-treat condition, presenting a significant clinical challenge. This study aims to investigate the protective mechanisms of Dexmedetomidine (Dex) against I/R injury, with a particular focus on its role in regulating autophagy activity in intestinal neurons and maintaining mitochondrial homeostasis. Experimental findings demonstrate that Dex can mitigate intestinal damage induced by I/R through the modulation of autophagy activity and mitochondrial function in intestinal neurons by suppressing the expression of Nupr1. This discovery sheds light on a new molecular mechanism underlying the potential efficacy of Dex in treating intestinal I/R injury, offering valuable insights for clinical therapy.

**Supplementary Information:**

The online version contains supplementary material available at 10.1186/s10020-024-00952-2.

## Introduction

Intestinal ischemia/reperfusion injury (I/R) is a common and severe clinical condition that occurs during cardiovascular surgeries, intestinal injuries, and other scenarios, potentially leading to tissue necrosis, inflammatory reactions, and cellular damage, ultimately posing risks such as sepsis and even endangering patients’ lives (Cai et al. 2022; Lin et al. 2021; Liu et al. 2023). During the I/R process, damaged intestinal tissues must cope with the complexities arising from the duration of ischemia and the reperfusion phase, involving diverse cellular biology and immune mechanisms (Karhausen et al. 2018). Ischemic tolerance time refers to the duration tissues or organs can endure under ischemic conditions. When tissues or organs are under ischemic conditions, their cells begin to suffer from inadequate oxygen supply, leading to abnormal cell metabolism and progressive cell damage. The length of ischemic tolerance time directly impacts the severity of ischemia-reperfusion injury. In early animal studies, the ileum was found to have shorter ischemic tolerance time compared to the jejunum, suggesting an ischemic tolerance time transfer of 1 h for the ileum microvasculature. Clinically, the ileum segment can tolerate ischemia well within 2 h (Farber et al. 1999; Chen et al. 2013). Hence, the quest for novel therapeutic approaches that can shield intestinal tissues from I/R injuries remains a focal point of current research efforts (Cai et al. 2022; Hu et al. 2022a, b; Zhang et al. 2023).

Intestinal neurons play a crucial role in physiological regulation and are significantly impacted by I/R injury (Agirman et al. 2021; Jacobson et al. 2021; Stockinger et al. 2021). Autophagy is a cellular process of self-degradation that helps maintain cellular homeostasis by eliminating damaged organelles and proteins. Studies suggest that during intestinal I/R injury, cellular autophagic activity may be impaired, leading to increased cellular oxidative stress and apoptosis (Shen et al. 2023; Li et al. 2023). Additionally, mitochondria serve as vital energy-producing organelles within cells, and their dysfunction can exacerbate the damage (Leite et al. 2023; Pereira et al. 2024). Therefore, ensuring the normal levels of autophagic activity in intestinal neurons and mitochondrial homeostasis is crucial for protecting against I/R injury (Klionsky et al. 2021).

Dexmedetomidine (Dex) is a widely used drug for anesthesia and pain management, recently found to have protective effects in various diseases like myocardial infarction and stroke (Berger et al. 2023; Bermejo et al. 2022). DEX is a highly selective α2 adrenergic receptor agonist commonly used for sedation and anesthesia. Studies have also highlighted DEX’s impact on ischemia-reperfusion injury, particularly in the intestines. Previous research has established that Dex alleviates renal ischemia-reperfusion injury and inflammation mediated by iron toxicity by inhibiting ACSL4 through α2-AR (Tao et al. 2022). Dex interacts with α2-AR in myocardial cells, enhancing the protective role of BK in cardiac I/R injury, suggesting that the modulation of endogenous cardiac protective factors may play a crucial role in Dex-induced cardioprotection (Zhan et al. 2022). Moreover, DEX suppresses PDIA3 by activating α2-AR, thus inhibiting inflammation, endoplasmic reticulum stress-dependent apoptosis, and oxidative stress induced by intestinal I/R in mice. Furthermore, Dex inhibits the TLR4/MyD88/NF-κB signaling pathway, reducing intestinal I/R injury in rats and OGD/R damage in Caco-2 cells (Yang et al. 2021).

This study hypothesizes that DEX may exert its effects in intestinal I/R injury through the regulation of Nupr1. Nupr1 is a protein closely associated with cellular autophagy and stress response, and its regulation may have significant impacts on neuronal autophagy and mitochondrial function (Fan et al. 2022; Zhan et al. 2022; Liu and Costa 2022). Through experiments and data analysis, we seek to elucidate the impact of DEX on intestinal I/R injury through the Nupr1-mediated mechanism, aiming to provide novel molecular targets for treatment. To validate this hypothesis, a series of experiments were conducted, including high-throughput transcriptome sequencing and single-cell RNA sequencing analysis of rat ileal tissue samples (Zhu et al. 2022; Wen et al. 2023). By studying the differential expression genes among different groups and their functional enrichment analysis, we identified key genes related to autophagy and mitochondrial homeostasis. This study aims to reveal the protective mechanism of DEX against intestinal I/R injury, particularly its regulation of neuronal autophagy and mitochondrial homeostasis through Nupr1 modulation.

## Materials and methods

### Transcriptome high-throughput sequencing and data analysis

We collected intestinal tissue samples from Sham group (*n* = 3), I/R group (*n* = 3), and Dex group (*n* = 3) of rats for transcriptome high-throughput sequencing. The specific steps were as follows: Total RNA from each sample was extracted using Trizol reagent (T9424, Sigma-Aldrich, USA) following the manufacturer’s instructions. RNA concentration, purity, and integrity were assessed using the Qubit^®^2.0 Fluorometer^®^ (Life Technologies, USA) with the Qubit^®^ RNA analysis kit, Nanodrop spectrophotometer (IMPLEN, USA), and Bioanalyzer 2100 system with the RNA Nano 6000 analysis kit (Agilent Technologies, USA), respectively. The A260/280 ratio was maintained between 1.8 and 2.0. A total of 3 µg of RNA per sample was used as input material for RNA sample preparation. The NEBNext^®^ UltraTM RNA directional library preparation kit for Illumina (E7760S, Gene Corporation, China) was employed to generate cDNA libraries, and their quality was assessed on the Agilent Bioanalyzer 2100 system. Subsequently, the indexed samples were clustered using the TruSeq PE Cluster Kit v3 cBot HS (Illumina) on the cBot cluster generation system following the manufacturer’s protocols. Library preparation was sequenced on the Illumina HiSeq 550 platform, yielding 125 bp/150 bp paired-end reads (Hong et al. 2020).

### Quality control and differential analysis of sequencing data

The quality of paired-end reads in the raw sequencing data was assessed using FastQC (v0.11.8) software. Preprocessing of the raw data was conducted with Cutadapt (v1.18) to remove Illumina sequencing adapters and poly(A) tail sequences. Reads with N content exceeding 5% were filtered out using a Perl script. The FASTX Toolkit (v0.0.13) software was utilized to extract reads with a minimum base quality of 20, accounting for 70% of the sequence length. The BBMap (v39.01) software was employed for the correction of paired-end sequences. Subsequently, the filtered high-quality read fragments were aligned with the human reference genome using hisat2 (v0.7.12) software.

For the analysis of differentially expressed genes (DEGs) between the Sham and I/R groups, as well as the I/R and Dex groups, based on high-throughput transcriptome sequencing data, the R package limma was employed with selection criteria of |logFC| > 2 and *P* < 0.05, as described in a previous study (Wang et al. 2022). The intersection genes were obtained by taking the intersection of the two differential analysis results using opposite trends.

### Gene functional enrichment analysis

To further elucidate the specific functions of the intersection genes, the R package clusterProfiler (Yu et al. 2012) was used with a significance threshold of *P* < 0.05. Enrichment analyses for Gene Ontology (GO) and Kyoto Encyclopedia of Genes and Genomes (KEGG) pathways were performed, including biological processes (BP), cellular components (CC), and molecular functions (MF). Bar charts and cluster dendrograms were generated for visualization.

### scRNA-seq data analysis

After obtaining the expression matrix using the official 10× Genomics software Cell Ranger, cells were selected based on the criteria: expressing a gene count (nFeature_RNA) > 200 and < 5000, RNA molecule count (nCount_RNA) > 1000 and < 20,000, and mitochondrial gene percentage (percent.mt) < 25. The quality of the filtered data was assessed through the analysis of a unique molecular identifier (UMI) and gene correlation (Aran et al. 2019).

Further analyses included principal component analysis (PCA) on the top 2000 variably expressed genes, JackStrawPlot and ElbowPlot functions were then used to determine the selection of principal components for downstream analysis. The Seurat FindClusters function was utilized to identify main cell subpopulations with a resolution set at 0.5. Subsequently, the UMAP algorithm was employed for non-linear dimensionality reduction and two-dimensional visualization of the scRNA-seq sequencing data. Marker genes for various cell subtypes were identified using the find marker genes function from the scCATCH package, followed by manual cell type annotation based on known cell marker genes (Shao et al. 2020).

### Cell culture and grouping

Intestinal neurons were obtained following the protocol outlined in a previous study. The cells were resuspended in modified N2 medium and cultured on coverslips coated with poly-D-lysine and laminin to support neuronal growth and differentiation (Nezami et al. 2014).

To simulate intestinal ischemia-reperfusion (I/R) injury, cells were first subjected to hypoxic conditions in an anaerobic culture chamber for 4 h, followed by a 4-hour recovery period under normal oxygen conditions. Based on the known Nupr1 sequence in NCBI, Shanghai Hanheng Biotechnology Co., Ltd. (Shanghai, China) was commissioned to construct oe-NC and oe-Nupr1 into the lentiviral vector pHBLV-CMV-MCS-EF1-Puromycin. Additionally, sh-NC and sh-Nupr1 were constructed into the silencing vector pHBLV-U6-MCS-PGK-Puromycin (LV019, Shanghai Hanheng Biotechnology Co., Ltd., URL). Cells in logarithmic growth phase were dissociated with trypsin and seeded at a density of 1 × 10^5^ cells per well in 6-well plates. After standard cultivation for 24 h, when cell confluence reached approximately 75%, viral particles (MOI = 10, working titer around 5 × 10^6^ TU/mL) along with 5 µg/mL polybrene (Merck, TR-1003, USA) were added to the medium for infection. Cell grouping is detailed in Table 1.

**Table 1:** In vitro cell experimental groups

Group	Treatment Description
Control	Untreated IEC-6 cells
Model	IEC-6 cells cultured under hypoxia for 4 h
Model + Dex	IEC-6 cells cultured under hypoxia for 4 h, treated with 2 µM Dex for 1 h
Model + Dex + oe NC	IEC-6 cells infected with oe NC lentivirus, cultured under hypoxia for 4 h, treated with 2 µM Dex for 1 h
Model + Dex + oe Nupr1	IEC-6 cells infected with oe Nupr1 lentivirus, cultured under hypoxia for 4 h, treated with 2 µM Dex for 1 h
Model + sh-NC	IEC-6 cells infected with sh-NC lentivirus, cultured under hypoxia for 4 h
Model + sh-Nupr1	IEC-6 cells infected with sh-Nupr1 lentivirus, cultured under hypoxia for 4 h

### Immunofluorescence assay

Cells were rinsed with PBS and fixed with 4% paraformaldehyde for 15 min. Subsequently, cell permeabilization was performed with 0.2% Triton X-100 in PBS for 5 min at room temperature. After permeabilization, block nonspecific binding by incubating the samples with 5% bovine serum albumin (BSA) or 10% normal goat serum at room temperature for 30 min. Cells were then incubated overnight at 4 °C with primary antibodies against LC3B (1:200, ab48394; Abcam) or p62 (1:200, P0067; Sigma-Aldrich). Following PBS washes, cells treated with anti-LC3B were incubated at room temperature for 1 h with Alexa Fluor^®^ 488 secondary antibody (1:500, ab150113; Abcam), while cells treated with anti-p62 were incubated at room temperature for 1 h with Alexa Fluor^®^ 647 secondary antibody (1:500, ab150115; Abcam). Nuclear staining was achieved using DAPI (Catalog No.: C0065, Beijing Soleibao Technology Co., Ltd., Beijing, China). Specimens were captured at 400-fold magnification using an inverted Olympus FV1000 laser scanning confocal microscope (Olympus). Fluorescence intensity was measured using ImageJ software to analyze the average fluorescence of randomly selected regions (Sun et al. 2022).

### Immunohistochemistry experiment

Intestinal tissue Sect. (4 μm thick) for immunohistochemistry were deparaffinized in xylene and hydrated in decreasing concentrations of ethanol. Subsequently, the tissue was subjected to antigen retrieval in citrate buffer (pH 6.0) at 95 ºC for 15 min. Endogenous peroxidase activity was blocked with a 3% H_2_O_2_ solution at 25 ºC for 10 min. Then, block nonspecific binding by incubating with 5% bovine serum albumin (BSA) or 10% normal goat serum at 25 °C for 30 min. The sections were then incubated with primary antibody at 25 ºC for 2 h, washed three times with phosphate-buffered saline (PBS), and incubated with secondary antibody (1:10000, BA1056, Boster, Wuhan, China) at 25 ºC for 20 min. Following this, the sections were incubated with DAB substrate for 5–10 min. Antibodies against LC3B (ab48394, 1:800, Abcam) and p62 (1:450, P0067, Sigma-Aldrich) were utilized. The expression of the relevant proteins was evaluated by measuring the percentage of positively stained cells (Wang et al. 2021; Liu et al. 2019).

### Detection of gene expression by RT-qPCR

Total RNA from intestinal neuron cells and tissues was extracted using Trizol reagent (15596026, Invitrogen, Thermo Fisher Scientific, USA). The concentration and purity of the extracted total RNA were examined using a Nanodrop 2000 spectrophotometer (1011U, Nanodrop, USA). The RNA was reverse-transcribed into cDNA following the instructions of the PrimeScript RT reagent Kit (RR047A, Takara, Japan) at 37 °C for 30–50 min and 85 °C for 5 s. RT-qPCR was performed using the Fast SYBR Green PCR Master Mix (RR820A, Takara, Japan) with the ABI PRISM 7300 RT-PCR system (Applied Biosystems, Thermo Fisher Scientific, USA). The reaction conditions included an initial denaturation at 95 °C for 5 min, followed by 40 cycles of denaturation at 95 °C for 30 s, annealing at 57 °C for 30 s, and extension at 72 °C for 30 s. Three replicates were set for each sample, and GAPDH was used as an internal control. The gene relative expression levels were analyzed using the 2^−ΔΔCt^ method, where ΔΔCt = (mean Ct value of the target gene in the experimental group - mean Ct value of the reference gene in the experimental group) - (mean Ct value of the target gene in the control group - mean Ct value of the reference gene in the control group). The experiment was repeated three times. The primers were designed and synthesized by Shanghai Sangon Biotech Company as shown in Table S1.

### Western blot

Cells from each group were collected and lysed on ice for 30 min at 4℃ with RIPA lysis buffer containing 1% PMSF (P0013B, Biyuntian, Shanghai, China), followed by centrifugation at 14,000 g. The supernatant was collected after centrifugation. The protein concentration of the samples was determined using the BCA method (P0012S, Biyuntian, Shanghai, China). 50 µg of protein was denatured by boiling for 10 min at 100℃ after adding an appropriate amount of 5× loading buffer. The denatured proteins were loaded onto the gel for electrophoresis using separating and concentrating gels. After electrophoresis, proteins were transferred to a PVDF membrane. The PVDF membrane was blocked in 5% non-fat milk powder at room temperature for 1 h, followed by overnight incubation at 4℃ with primary antibodies: rabbit anti-LC3-II (ab192890, Abcam, USA), rabbit anti-P62 (ab314504, Abcam, USA), rabbit anti-Nupr1 (orb459164, Wuhan BOOSE Biotechnology Co., Ltd.), and rabbit anti-GAPDH (ab181602, Abcam, USA). GAPDH was used as an internal control. After washing with PBST at room temperature, the membrane was incubated with Horseradish Peroxidase (HRP)-conjugated goat anti-rabbit IgG secondary antibody (1:10000, BA1056, Boster, Wuhan, China) for 1 h. The membrane was then washed six times for 5 min each with PBST. ECL substrate solution (AR1172, Boster, Wuhan, China) was evenly applied to the membrane, followed by exposure in an imaging system (Amersham Imager 600, USA) for chemiluminescence detection (Salem et al. 2019; Shu et al. 2011). Image J software was used for grayscale analysis. The experiment was repeated three times.

### Staining with hoechst 33,342

The cells in the logarithmic growth phase were digested with trypsin to prepare cell suspensions. After centrifugation, the cells were seeded in 24-well plates with a seeding density of 5000 cells/500 µL. Following a 24-hour incubation, cell growth and uniform distribution were observed under an inverted microscope. Subsequently, the culture medium was aspirated when cell growth was satisfactory, and the control group received a complete medium while the I/R and Dex groups were subjected to respective interventions as previously described, followed by removal of the supernatant. The cell monolayers were washed twice with PBS. The cells were fixed with 3.7% formaldehyde, with 300 µL added per well for 15 min, followed by two washes with PBS. Hoechst 33,342 working solution (C1029, Biosharp, Shanghai, China) was added to each well (200 µL) for cell staining, which was carried out at room temperature in the dark for 15 min. The cell monolayers were then washed twice with PBS. Finally, the slides were mounted with an anti-fade mounting medium (P0126, Biosharp, Shanghai, China) and observed under a fluorescent inverted microscope (DMI4000 B, Leica Microsystems, Germany) for staining evaluation and imaging (Zhang et al. 2021).

### Observation of cellular ultrastructure with transmission electron microscopy

Intestinal tissues were first fixed overnight at 4℃ in 2% glutaraldehyde (111-30-8, Aike Reagent, China), followed by a 1-hour fixation with 1% osmium tetroxide (OsO_4_) (115355, Aike Reagent, China). The tissues were dehydrated in an ethanol series and embedded in epoxy resin. Thin sections were cut with a microtome and placed on copper grids with support films. After negative staining, the tissues were observed using a transmission electron microscope (JEM-1010; JEOL, Tokyo, Japan) for ultrastructural analysis (Zhang et al. 2021).

### Mitochondrial function assessment

Mitochondrial membrane potential changes were assessed using the MMP assay kit (P0338S, Beyotime, China). After 24 h of cell treatment, cell culture supernatant was collected and centrifuged. Following the removal of the supernatant, the cells were stained according to the instructions provided with the kit. Normal MMP was detected using a flow cytometer (660585, BD, USA). The Flowjo software was utilized for data analysis to obtain the characteristics of MMP changes in each group of cells. Each experiment was repeated three times.

The levels of MDA and SOD enzyme activity in rat intestinal tissues were evaluated using commercial assay kits (a003-1 and a001-3-2, Nanjing Jiancheng Bioengineering Institute, Nanjing, China). Following the kit instructions, intestinal tissues were homogenized, and quantitative analysis was performed using the BCA protein assay. Subsequently, the total levels of MDA and SOD activity in the tissue homogenate were calculated.

Cellular ROS levels were detected using the DCFH-DA probe (HY-D0940, MCE, USA). H2DCFDA was dissolved in DMSO to obtain a 10 mM stock solution and further diluted before use. Cells were incubated at 37 °C in the dark with a PBS solution containing 5 µM H2DCFDA for 30 min, followed by washing with PBS, addition of fresh culture medium, and immediate imaging using a fluorescence microscope (Zhang et al. 2021).

### Flow cytometry analysis of cell apoptosis

Cell apoptosis analysis was performed using the Annexin V-FITC/PI apoptosis detection kit (AB_2869082, BD Biosciences, USA). For the analysis, cell culture supernatant was collected and cells were digested, neutralized, and washed three times with DPBS followed by centrifugation at 1200 rpm for 5 min. After cell counting, cells were resuspended in 100 µl of 1× annexin-binding buffer, and 5 µl of Annexin V-FITC and 1 µl of 100 µg/ml PI working solution were added separately. The cells were then incubated at room temperature in the dark for 15 min. Following the incubation, each tube received 400 µl of 1× annexin-binding buffer, mixed well, and analyzed on a C6 flow cytometer (Feng et al. 2020).

### Construction of animal models

Eighty healthy male Sprague-Dawley (SD) rats (hnslkjd001, Hubei Slake Jinda Experimental Animal Co., Ltd., China), aged 7–9 weeks with a weight ranging from 210 g to 300 g, were randomly divided into 8 groups, with 10 animals in each group (Table 2 for groupings and treatments). Based on the known Nupr1 sequence in NCBI, Shanghai Hengheng Biological Engineering Co., Ltd. (Shanghai, China) was commissioned to construct the overexpression nc, oe Nupr1 lentiviral vector pHBLV-CMV-MCS-EF1-Puromycin, and the knockdown sh-NC, sh-Nupr1 silencing vector pHBLV-U6-MCS-PGK-Puromycin (LV019, Shanghai Hengheng Biological Engineering Co., Ltd.). The rats were injected with lentivirus via tail vein at a dose of 5 × 10^6^ TU (Transduction Units) per rat, followed by subsequent procedures after 48 h. A 12-hour fasting period and free access to water were provided preoperatively.

**Table 2:** In vivo rat grouping

Group	Treatment Description
Sham	Sham-operated rats, without SMA occlusion
I/R	Rats subjected to ischemia-reperfusion treatment
I/R + Dex1	Rats subjected to ischemia-reperfusion, continuously infused with Dexmedetomidine at 2.5 µg/kg/h for 1 h
I/R + Dex2	Rats subjected to ischemia-reperfusion, continuously infused with Dexmedetomidine at 5.0 µg/kg/h for 1 h
I/R + Dex + oe-NC	Rats subjected to ischemia-reperfusion, continuously infused with Dexmedetomidine at 5.0 µg/kg/h for 1 h, followed by tail vein injection of oe-NC lentivirus
I/R + Dex + oe-Nupr1	Rats subjected to ischemia-reperfusion, continuously infused with Dexmedetomidine at 5.0 µg/kg/h for 1 h, followed by tail vein injection of oe-Nupr1 lentivirus
I/R + sh-NC	Rats subjected to ischemia-reperfusion, followed by tail vein injection of sh-NC lentivirus
I/R + sh-Nupr1	Rats subjected to ischemia-reperfusion, followed by tail vein injection of sh-Nupr1 lentivirus

The rats were anesthetized by intraperitoneal injection of 1% pentobarbital sodium (30 mg/kg) (CAS 57-30-7, Shanghai Xinyasi Pharmaceutical, China) and positioned supine on the operating table, with the abdominal area sterilized with polyvinylpyrrolidone iodine under warm white light to maintain rectal temperature between 37 ~ 38℃. A 1–1.5 cm midline incision was made along the abdominal wall. The intestinal loops were gradually freed starting from the ileocecal junction to expose the superior mesenteric artery (SMA) and occlude the blood flow using non-traumatic microvascular clamps. Successful establishment of intestinal ischemia was confirmed by dark red coloration of the intestines, cessation of SMA pulsation, and disappearance of peristalsis. The ischemia onset time was recorded, and then the intestines were repositioned, the abdominal cavity was closed, and the incision was sutured. After 1 h of ischemia, the abdominal cavity was reopened, and the vascular clamps were released to restore blood flow and allow for intestinal reperfusion. Observation of the intestinal tissue transitioning from dark red to bright red color, restoration of SMA pulsation, and peristalsis indicated successful reperfusion, with the reperfusion start time documented.

During the intestinal I/R period, rats in all groups received continuous tail vein infusion of 0.9% sodium chloride solution at a rate of 1.5mL/h. Subsequently, a 5% cefoperazone sodium injection (50 mg/kg) (HY-B0210, MCE, USA) was administered to prevent further infections, the incision was closed, and reperfusion continued for 2 h (Gubernatorova et al. 2016; Sang et al. 2021). For the Sham group, rats were anesthetized and then underwent continuous intravenous infusion of normal saline at a rate of 1.5mL/h for 1 h. The abdominal cavity was then opened to expose the intestines without occluding the SMA. In contrast, the I/R group underwent SMA occlusion for 60 min on top of the Sham procedure, followed by 120 min of reperfusion. Rats in the I/R + Dex1 group received continuous intravenous Dex infusion at a rate of 2.5 µg/kg/h before SMA occlusion. After pre-treatment, the abdominal cavity was opened to expose the intestines, followed by 60-minute SMA occlusion and 120-minute reperfusion. The rats in the I/R + Dex2 group received continuous intravenous Dex infusion at a rate of 5.0 µg/kg/h before SMA occlusion, with subsequent procedures identical to the I/R + Dex1 group.

Following the reperfusion period, the rats were anesthetized again with pentobarbital sodium for sample collection. Blood was obtained via the abdominal aorta, centrifuged to collect the upper serum layer, and stored in a -80 °C freezer. After euthanasia, the cecum was identified as a landmark, and intestinal tissue was collected 5 cm above it. Initially, a 2 cm segment of the small intestine was rinsed with saline, dried, weighed on an electronic scale, recorded as wet weight, placed in an 80 °C oven for 24 h, then reweighed for dry weight. Subsequently, another 2 cm section of intestinal tissue was cleansed of blood residues with ice-cold saline, and fixed in a 10% neutral formalin solution. Next, a 5 cm portion of the remaining small intestine was dissected, surrounding mesentery and lymph nodes were cleaned in pre-chilled PBS, feces were removed from the intestines, and several 1 cm segments of the jejunum were reserved. These segments were individually placed in labeled cryotubes, and stored in a liquid nitrogen tank, and all samples were later transferred into labeled sample bags and finally stored in a -80 °C freezer.

Upon completion of sample collection, all rats were euthanized using cervical dislocation (Feng et al. 2020).

### H&E staining

Tissue samples were fixed in 4% paraformaldehyde (30525-89-4, Sigma-Aldrich, USA) for 30–50 min. After deparaffinization and hydration, paraffin-embedded sections were stained with hematoxylin-eosin (15086-94-9, Sigma-Aldrich, USA) for 5 min, followed by differentiation in 1% hydrochloric acid and subsequent eosin staining for 3 min. Subsequently, the sections underwent dehydration, clearing, and mounting before being observed under a microscope. Intestinal mucosal damage was assessed using the Chiu scoring system: 0, normal intestinal mucosa villi; 1, presence of subepithelial spaces within the villus axis with accompanying capillary congestion; 2, upward migration of the epithelium from the lamina propria and enlargement of the subepithelial space; 3, significant elevation of the intestinal mucosa epithelium, collapse of villi sideways, and tip villi shedding; 4, collapse of villi and lamina propria, exposure of dilated capillaries, and increased cellular components of the lamina propria; 5, lamina propria disintegration or digestion, bleeding, or ulcer formation (Feng et al. 2020).

### TUNEL staining

TUNEL staining to detect cell apoptosis was performed using a one-step TUNEL apoptosis detection kit (C1086, Beyotime, Shanghai, China). Initially, sections were treated with 0.1% Triton X-100 (Beyotime, Shanghai, China) at 4 °C for 3 min and incubated with proteinase K for 15 min, followed by the addition of 50 µL of TUNEL working solution. After 1 h of incubation at 37 °C in the dark, the plate was sealed with an anti-fade mounting solution, and fluorescence intensity was observed under a fluorescence microscope (DMI4000 B, Leica Microsystems, Germany). Five high-power fields were randomly selected to calculate the number of TUNEL-positive cells (Venkatesan et al. 2013).

### ROS level detection

The intestinal lumen was washed with PBS until free of any content, and excess water in the intestinal tissue was removed by blotting with absorbent paper. The tissue was then embedded in an OCT embedding compound. The embedded intestinal tissue was sectioned to a thickness of 4 μm using a cryostat. The cryosections were rinsed in PBS to remove any impurities, and a circle was drawn around the tissue using a histochemical pen. Subsequently, 100 µL of DHE (7.5 mM, MedChemExpress, China) was added within the circle and allowed to react at room temperature in the dark for 30 min. Following incubation, the sections were washed with PBS and mounted with DAPI stain. Fluorescent images were captured using an inverted fluorescence microscope with excitation and emission wavelengths of 480 nm and 580 nm, respectively. Ten random fields were selected from each sample, and the percentage of DHE-positive cells was calculated (Zheng et al. 2020).

### ELISA experiment

Blood samples extracted from the abdominal aorta of rats were centrifuged at 3000 rpm for 15 min at 4 °C following overnight incubation at 4 °C. Proteins from intestinal tissues were extracted using homogenization and sonication methods. Monoclonal antibodies against IL-6 and TNF-α were coated onto a 96-well microplate following the instructions provided in the Rat IL-6 ELISA kit (ab234570, Abcam, USA) and Rat TNF-α ELISA kit (ab236712, Abcam, USA). The plates were then incubated overnight at 4 °C, followed by a 1-hour blocking step at room temperature, and washed with PBS. Subsequent steps were carried out according to the kit instructions. The optical density (OD) values were measured at a wavelength of 450 nm using a microplate reader (A51119500C, Thermo Fisher, USA) (Ma et al. 2019).

### Statistical analysis

The data were derived from at least three independent experiments, and the results are presented as mean ± standard deviation (Mean ± SD). For comparisons between the two groups, a two-sample independent t-test was employed. For comparisons involving three or more groups, a one-way analysis of variance (ANOVA) was conducted. In cases where the ANOVA revealed significant differences, post hoc Tukey’s Honestly Significant Difference (HSD) test was performed to compare differences between individual groups. Non-normally distributed or inhomogeneous variance data were analyzed using the Mann-Whitney U test or the Kruskal-Wallis H test. All statistical analyses were carried out using GraphPad Prism 9 (GraphPad Software, Inc.) and the R programming language. The significance level for all tests was set at 0.05, and a two-tailed p-value less than 0.05 was considered statistically significant.

## Result

### Dex regulates autophagy activity and mitochondrial homeostasis to alleviate intestinal I/R injury

Dex has been demonstrated to have a supportive role in protecting against I/R injury. While Dex can be utilized in treating I/R, its molecular mechanisms remain incompletely understood (Zhang et al. 2020). Therefore, an intestinal I/R rat model was established, and the protective effects of Dex on intestinal injury were investigated. A total of 40 SD rats were divided into four groups for experimentation: control group, I/R model group, Dex1 treatment group, and Dex2 treatment group for I/R.

**Fig. 1:**
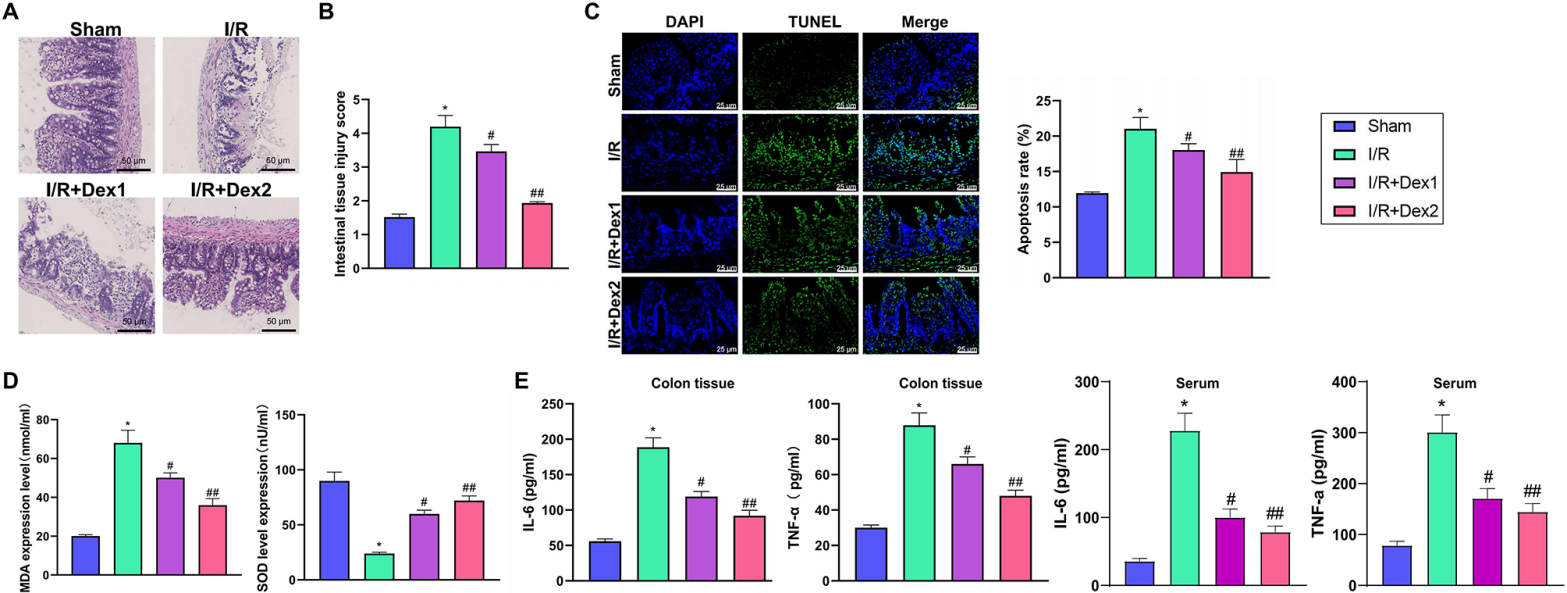
Protective effects of Dex in an intestinal I/R rat model. Note: (**A**) Histological examination using H&E stain shows differences in the degree of intestinal tissue damage among the Sham group, I/R model group, and the I/R + Dex1 and I/R + Dex2 treatment groups in rats; (**B**) Tissue pathological damage scores (Chiu’s score) assess the extent of intestinal tissue damage in the Sham group, I/R model group, and the I/R + Dex1 and I/R + Dex2 treatment groups of rats; (**C**) TUNEL staining results compare the number of apoptotic cells in the intestinal tissues of rats in the Sham group, I/R model group, and the I/R + Dex1 and I/R + Dex2 treatment groups; (**D**) Comparison of MDA and SOD levels in intestinal tissues of rats in the Sham group, I/R model group, and the I/R + Dex1 and I/R + Dex2 treatment groups; (**E**) ELISA experimental results demonstrate differences in TNF-α and IL-6 levels in the serum and colonic tissue of rats in the Sham group, I/R model group, and the I/R + Dex1 and I/R + Dex2 treatment groups. *P<0.05 compared to Sham; #*P*<0.05, ##*P*<0.01 compared to I/R. *N* = 10

Histological examination of intestinal tissue samples through H&E staining revealed the nuclei stained in blue-purple and the cytoplasm in pink. Under the microscope, the Sham group rats exhibited normal intestinal structure with intact villi and glands, whereas the I/R group showed noticeable villous edema, shedding, severe glandular damage, breakdown of the lamina propria, significant infiltration of neutrophils, and some areas of bleeding. The I/R + Dex1 group exhibited milder mucosal edema and shedding, desquamation of epithelial cells, and damaged glands with minimal neutrophil infiltration in the lamina propria. The I/R + Dex2 group displayed significant improvements with minor changes in villous morphology, intact mucosa and glands, and no apparent neutrophil infiltration in the lamina propria (Fig. 1A). A comparison of intestinal tissue injury scores (Chiu’s score) between the Sham group and the I/R group showed a significant increase (*P* < 0.05) in the I/R group. In contrast, both I/R + Dex1 and I/R + Dex2 groups exhibited significantly reduced injury scores compared to the I/R group, with the trend being more pronounced in the I/R + Dex2 group (*P* < 0.05) (Fig. 1B). These results indicate that Dex plays a protective role in intestinal I/R injury.

The TUNEL assay is a commonly used method for detecting cellular apoptosis and can reflect the apoptotic status of cells. The results indicate that, compared to the Sham group, the rate of intestinal tissue cell apoptosis in the I/R group of rats significantly increased (*P* < 0.05). When comparing with the I/R group, the rates of cell apoptosis in the intestinal tissues of rats in the I/R + Dex1 and I/R + Dex2 groups were significantly reduced, with the trend being more prominent in the I/R + Dex2 group (*P* < 0.05) (Fig. 1C).

By measuring the levels of Malondialdehyde (MDA) concentration and total superoxide dismutase (SOD) activity in the intestinal mucosal tissues of each group, reflecting tissue oxidative stress levels, the results revealed that concerning the Sham group, the MDA levels in intestinal tissues of rats in the I/R group significantly increased (*P* < 0.05). In comparison to the I/R group, the I/R + Dex1 and I/R + Dex2 groups showed significant reductions in MDA levels in intestinal tissues, with the I/R + Dex2 group demonstrating a more pronounced trend (*P* < 0.05). Regarding the SOD activity levels in the intestinal tissues of rats, compared with the Sham group, the I/R group exhibited a significant decrease (*P* < 0.05). In contrast, when compared to the I/R group, both the I/R + Dex1 and I/R + Dex2 groups showed significantly increased SOD activity levels, with the I/R + Dex2 group displaying a more notable trend (*P* < 0.05) (Fig. 1D).

Serum and colonic tissue ELISA results showed that compared to the Sham group, levels of TNF-a in the serum and colonic tissue of rats in the I/R group significantly increased (*P* < 0.05). When comparing with the I/R group, both the I/R + Dex1 and I/R + Dex2 groups exhibited a significant decrease in TNF-a levels in the serum and colonic tissue, with the I/R + Dex2 group showing a more pronounced trend (*P* < 0.05). Furthermore, in comparison to the Sham group, levels of IL-6 in the serum and colonic tissue of rats in the I/R group significantly elevated (*P* < 0.05). Conversely, when compared to the I/R group, both the I/R + Dex1 and I/R + Dex2 groups showed a significant reduction in IL-6 levels in the serum and colonic tissue, with the I/R + Dex2 group demonstrating a more significant trend (*P* < 0.05) (Fig. 1E). These findings indicate that DEX effectively mitigates the inflammatory response and cellular apoptosis caused by intestinal ischemia-reperfusion injury, further supporting its potential therapeutic value in treating intestinal ischemia-reperfusion injury.

### Dex may improve intestinal I/R injury in rats by modulating autophagy activity and mitochondrial homeostasis

**Fig. 2:**
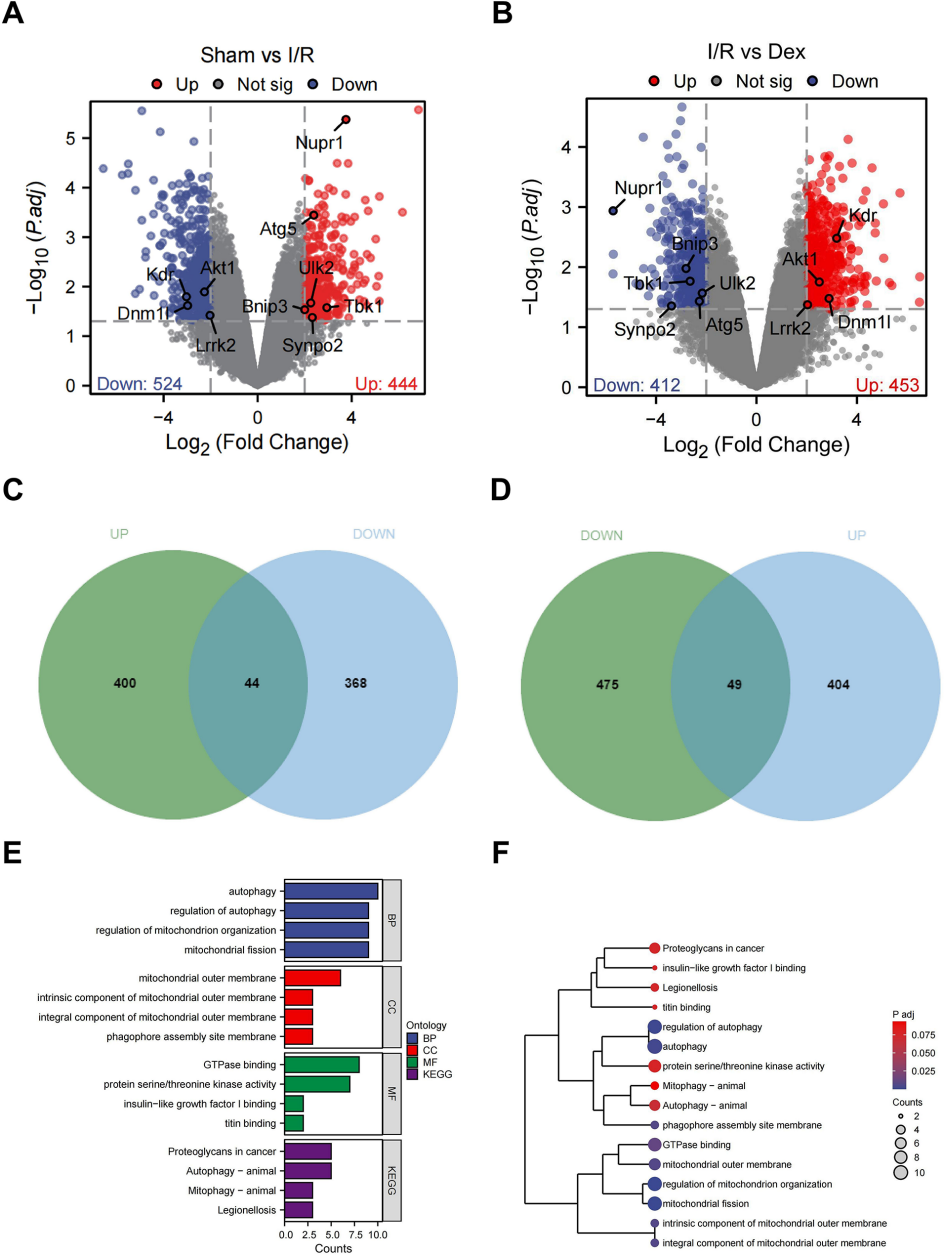
Mechanisms of Dex action analyzed by high-throughput RNA sequencing. Note: (**A**) Volcano plot of differential analysis between Sham group (*N* = 3) and I/R group (*N* = 3); (**B**) Volcano plot of differential analysis between I/R group (*N* = 3) and Dex group (*N* = 3). In Figures A and B, red represents upregulated genes, blue represents downregulated genes, gray represents genes with insignificant differences, and the black dashed box highlights the significant DEG Nupr1; (**C**) Venn diagram showing the intersection of upregulated genes in the I/R group (compared to Sham group) and downregulated genes in the Dex group (compared to I/R group); (**D**) Venn diagram showing the intersection of downregulated genes in the I/R group (compared to Sham group) and upregulated genes in the Dex group (compared to I/R group); (**E**) Bar graph of GO and KEGG enrichment analysis of all intersecting genes; (**F**) Dendrogram of GO and KEGG enrichment analysis of all intersecting genes

To elucidate the specific mechanisms by which Dex improves intestinal I/R injury in rats, we collected ileum tissue samples from Sham, I/R, and Dex groups for high-throughput RNA sequencing analysis. The sequencing data revealed that there were 967 DEGs between the Sham and I/R groups, with 444 upregulated and 524 downregulated (Fig. 2A); and 865 DEGs between the I/R and Dex groups, with 453 upregulated and 412 downregulated (Fig. 2B). By intersecting the two sets of differential analysis results with opposite trends, we identified 93 intersecting genes, among which 44 genes were upregulated in the I/R group (compared to the Sham group) and downregulated in the Dex group (compared to the I/R group) (Fig. 2C); while 49 genes were downregulated in the I/R group (compared to the Sham group) and upregulated in the Dex group (compared to the I/R group) (Fig. 2D).

The GO and KEGG analyses of the 93 intersecting genes revealed that these genes were mainly enriched in autophagy and mitochondrial homeostasis (Fig. 2E-F). Notably, Nupr1 showed the most significant differential expression among the Sham, I/R, and Dex groups (Fig. 2A-B). Therefore, we propose that Dex may improve intestinal I/R injury in rats by modulating autophagy activity and mitochondrial homeostasis, with Nupr1 being a key gene in this process.

### Cell clustering and distribution of Nupr1 expression across different cell types in single-cell RNA sequencing analysis

To determine the cellular role of Nupr1 in rat intestinal tissues, we obtained scRNA-seq data from three normal rat ileum samples using the GEO database (GSE244963) with the aim of analyzing the cellular heterogeneity of Nupr1 through single-cell transcriptomics.

Initially, we performed quality control and normalization of the data using the R package Seurat, filtering out low-quality cells and retaining cells with gene expression per cell (nFeature_RNA) between 200 and 5000, RNA counts per cell (nCount_RNA) between 1000 and 20,000, and a percentage of mitochondrial genes (percent.mt) less than 25%. Consequently, 28,242 single cells passed quality control (Figure S1A). Correlation analysis of the filtered data revealed a correlation coefficient of *r* = -0.15 between nCount_RNA and percent.mt, and a correlation coefficient of *r* = 0.95 between nCount_RNA and nFeature_RNA (Figure S1B), indicating good data quality post-filtering. Subsequently, highly variable genes were identified from the filtered cells, and the top 2000 variable genes were selected for downstream analysis (Figure S1C).

Next, we performed batch correction and integration of all samples, followed by PCA of the top 2000 variable genes using the RunPCA function. This analysis showed no apparent batch effects among the three samples, demonstrating their suitability for subsequent analyses (Figure S1D). Additionally, a heatmap of the top two principal components was generated using the DimHeatmap function (Figure S1E), highlighting the main contributing genes to these components (Figure S1F). Visualizing the importance of the first 50 principal components using the JackStrawPlot function revealed that the first 8 components had P-values below 0.05, indicating their significance in capturing information from the highly variable genes (Figure S1G). Furthermore, the ElbowPlot function identified a change in standard deviation at the 8th principal component (Figure S1H). Taken together, the results suggested that the first 8 principal components effectively represented the information contained in the selected highly variable genes and held significant analytical value. Consequently, we proceeded with UMAP clustering analysis using these 8 principal components.

**Fig. 3:**
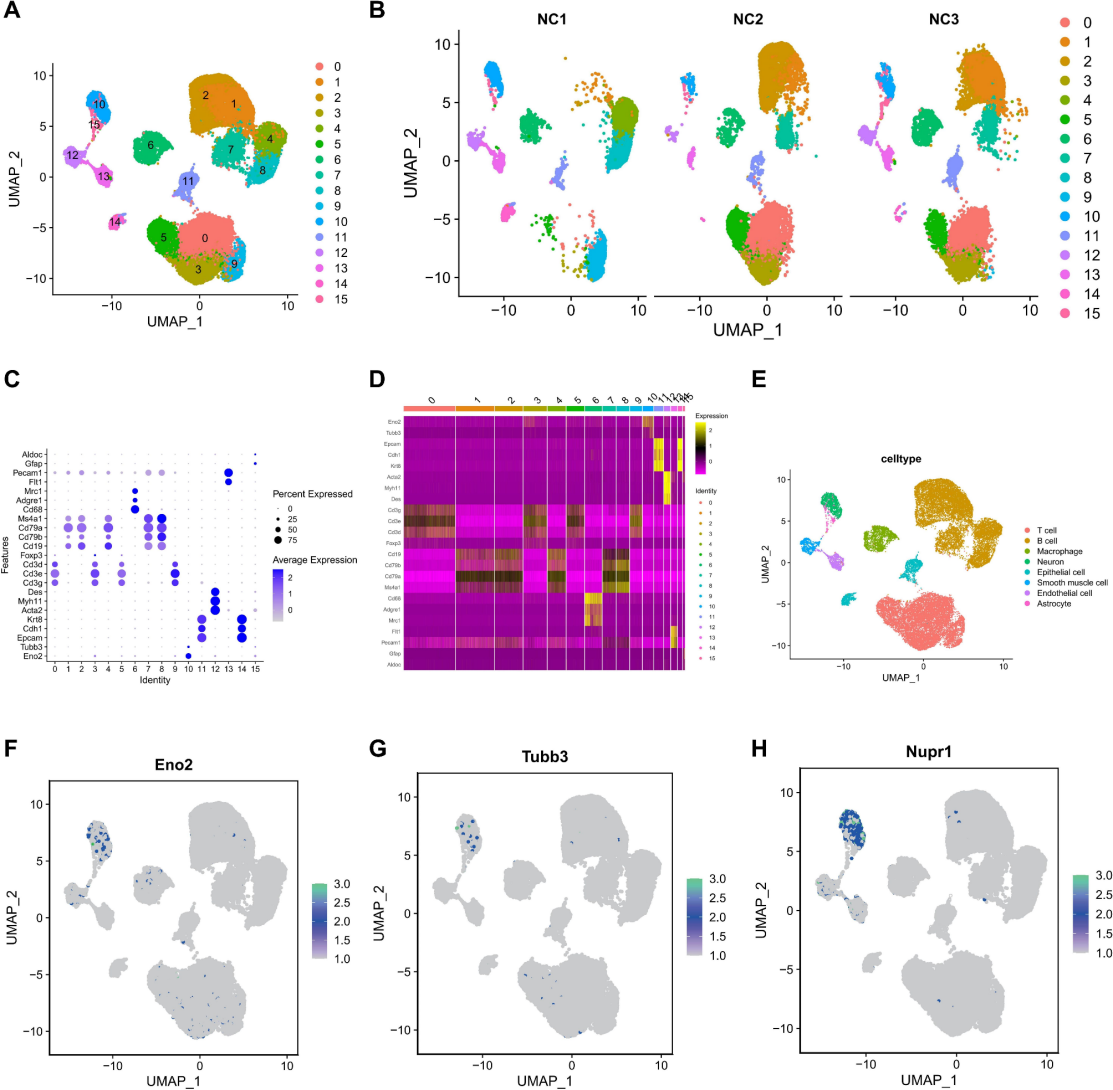
Single-cell atlas of Jejunum tissue samples from 3 healthy rats. Note: (**A**) UMAP analysis clustering all cells into 16 cell clusters, where each color represents a cluster; (**B**) Distribution of cell clusters from each sample origin; (**C**) Annotation of the 16 cell clusters into 8 cell types; (**D**) Dot plot showing the expression of marker genes in the 16 cell clusters; (**E**) Heatmap displaying the expression of marker genes in the 16 cell clusters; (**F**) Expression of intestinal neuron marker gene Eno2 in various cells; (**G**) Expression of intestinal neuron marker gene Tubb3 in various cells; (**H**) Expression of Nupr1 in various cells

Subsequently, the UMAP algorithm was employed to perform nonlinear dimensionality reduction on the top 8 principal components, clustering all cells into 16 cell clusters (Fig. 3A-B). Based on cell marker genes, these clusters were annotated into 8 major cell types, namely Neuron (Cluster 10, marked by Eno2 and Tubb3), Epithelial cell (Clusters 11 and 14, marked by Epcam, Cdh1, and Krt8), Smooth muscle cell (Cluster 12, marked by Acta2, Myh11, and Des), T cell (Clusters 0, 3, 5, and 9, marked by Cd3g, Cd3e, Cd3d, and Foxp3), B cell (Clusters 1, 2, 4, 7, and 8, marked by Cd19, Cd79b, Cd79a, and Ms4a1), Macrophage (Cluster 6, marked by Cd68, Adgre1, and Mrc1), Endothelial cell (Cluster 13, marked by Flt1 and Pecam1), and Astrocyte (Cluster 15, marked by Gfap and Aldoc) (Fig. 3C-E). It was observed that Nupr1 was predominantly expressed in neurons (Fig. 3F-H). Therefore, this study aims to further investigate the specific mechanism by which Dex improves autophagy activity and mitochondrial homeostasis by suppressing Nupr1 expression in intestinal neurons.

### Dex downregulates Nupr1 to Increase intestinal neuron autophagy activity and improve mitochondrial homeostasis

**Fig. 4:**
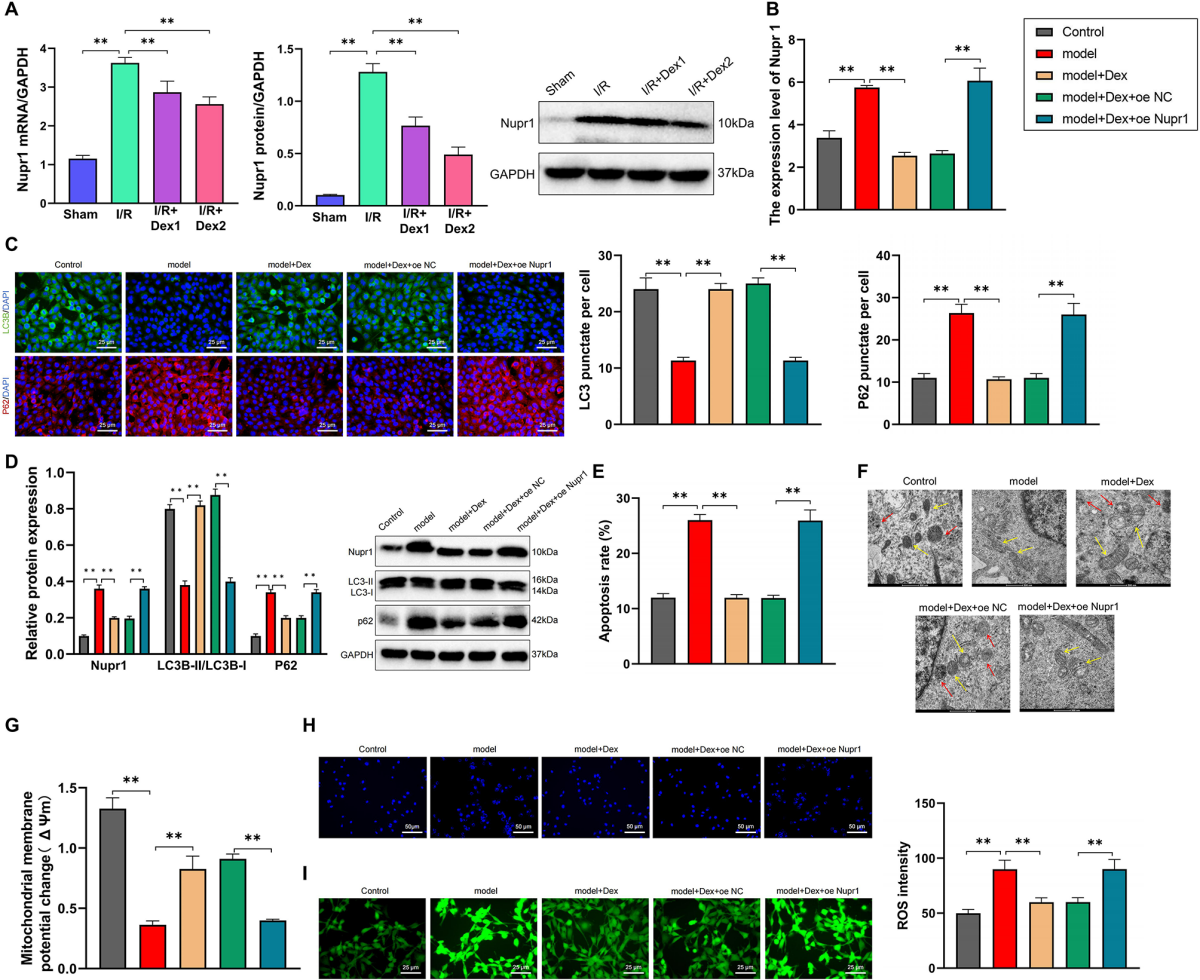
The impact of Dex on autophagy activity and mitochondrial function in intestinal neuron cell lines. Note: (**A**) Quantification of Nupr1 expression in the animal model using qPCR and Western blot; (**B**) Measurement of Nupr1 expression in cell models using qPCR; (**C**) Immunofluorescence detection of protein fluorescence levels of LC3B and p62 in control, model, model + Dex, model + Dex + oe NC, and model + Dex + oe Nupr1 groups; (**D**) Western blot analysis of Nupr1, LC3, and P62 protein expression in cell models of control, model, model + Dex, model + Dex + oe NC, and model + Dex + oe Nupr1. (**E**) Flow cytometry assessment of early apoptosis rate in different cell groups; (**F**) The observation of changes in the ultrastructure of cells in each group using transmission electron microscopy (with yellow arrows indicating mitochondria and red arrows indicating autophagosomes); (**G**) Measurement of mitochondrial membrane potential by detecting MMP; (**H**) Observation of apoptosis in cells of each group through Hoechst 33,342 staining; (**I**) Detection of ROS levels using H2DCFDA probe. *P<0.05, **P<0.01. *N* = 10, cell experiments were conducted in triplicate

In the animal model, the expression of Nupr1 was validated through qPCR experiments. Compared to the Sham group, the I/R group showed a significant increase in Nupr1 expression, while the expression levels of Nupr1 in the I/R + Dex1 and I/R + Dex2 groups were significantly decreased compared to the I/R group. The Western blot results were consistent with the qPCR results, indicating a notable upregulation of Nupr1 expression in the I/R group compared to the Sham group. Additionally, the expression levels of Nupr1 in the I/R + Dex1 and I/R + Dex2 groups were significantly reduced compared to the I/R group (*P* < 0.05) (Fig. 4A).

The cells were subjected to simulated I/R conditions and then treated with Dex (2 µM) for 24 h. qPCR experiments were conducted to measure the Nupr1 expression in the cell model groups. The model group exhibited a significant increase in Nupr1 expression compared to the control group, while the Nupr1 expression was significantly decreased in the model + Dex group compared to the model group. Moreover, the Nupr1 expression in the model + Dex + oe Nupr1 group was significantly elevated compared to the model + Dex + oe NC group (Fig. 4B). These findings indicate that Dex can influence the expression of Nupr1 in the intestine I/R model, and the overexpression of Nupr1 in the model + Dex + oe Nupr1 group was successful.

Immunofluorescence was utilized to detect the expression levels of LC3B and p62-related proteins in the cell models of each group. The results showed that compared to the control group, the model group exhibited a significant increase in P62 fluorescence intensity and a significant decrease in LC3B fluorescence intensity (*P* < 0.01). After Dex treatment, the P62 fluorescence intensity was significantly reduced, and the LC3B fluorescence intensity was markedly increased compared to the model group (*P* < 0.01). Furthermore, compared to the model + Dex group, the overexpression of Nupr1 in the model + Dex group resulted in a significant increase in P62 fluorescence intensity and a notable decrease in LC3B fluorescence intensity (*P* < 0.01) (Fig. 4C). Protein immunoblotting was conducted to detect the expression levels of Nupr1 and autophagy-related proteins in the cell models of each group. The results indicated that compared to the control group, the model group showed a significant increase in Nupr1 and P62 protein expression levels, along with a substantial decrease in the ratio of LC3II/LC3I expression (*P* < 0.01). Following Dex treatment, the Nupr1 and P62 protein expression levels were significantly reduced, while the ratio of LC3II/LC3I expression was markedly increased compared to the model group (*P* < 0.01). Notably, in the model + Dex group, the overexpression of Nupr1 led to a significant increase in Nupr1 and P62 protein expression levels and a considerable decrease in the ratio of LC3II/LC3I expression (*P* < 0.01) (Fig. 4D).

The Annexin-V/PI dual staining flow cytometry results showed a significant increase in the early apoptosis rate of cells in the model group compared to the control group (*P* < 0.01). However, after Dex treatment, the early apoptosis rate of Intestinal neuron cells decreased significantly compared to the model group (*P* < 0.01). In the model + Dex group, the overexpression of Nupr1 led to a significant increase in the early apoptosis rate of cells (*P* < 0.01) (Fig. 4E).

The transmission electron microscopy results showed that in the control group, intestinal neurons exhibited a small number of damaged mitochondria. In contrast, the model group displayed signficant mitochondrial morphological abnormalities, including swollen mitochondria, incomplete mitochondrial membranes, mitochondrial dissolution, and balloon-like degeneration, along with a decrease in autophagosomes. Compared to the model group, Dex treatment improved mitochondrial conditions and increased the number of autophagosomes. However, in the model + Dex group, overexpression of Nupr1 led to abnormal mitochondrial morphology and a reduction in autophagosomes (Fig. 4F). The mitochondrial membrane potential (∆Ψm) of Intestinal neuron cells in each group was measured using the MMP assay kit (JC-1) as per the instructions. The results showed membrane potentials of 1.33 ± 0.14, 0.37 ± 0.05, 0.85 ± 0.07, 0.91 ± 0.07, and 0.39 ± 0.05 for the respective groups. Compared to the control group, the MMP of cells in the model group significantly decreased (*P* < 0.05). However, Dex treatment led to a significant increase in the MMP of Intestinal neuron cells compared to the model group, while overexpression of Nupr1 in the model + Dex group resulted in a significant decrease in MMP (Fig. 4G). The Hoechst 33,342 staining results revealed that compared to the control group, the cells in the model group exhibited more pronounced apoptotic features, such as reduced cell numbers, nuclear condensation, nuclear displacement, chromatin condensation, and the formation of apoptotic bodies. However, upon addition of Dex treatment, as compared to the model group, there was a decrease in the apoptotic features of intestinal neurons. In the model + Dex group, overexpression of Nupr1 reduced the apoptotic features of cells significantly (Fig. 4H). H2DCFDA staining results showed that compared to the control group, the model group exhibited elevated levels of ROS, which were reduced after Dex treatment. However, in the model + Dex group with Nupr1 overexpression, ROS levels were increased (Liu et al. 2021) (Fig. 4I).

To investigate the impact of Nupr1 silencing on intestinal neuronal cell lines under intestinal ischemia-reperfusion (I/R) conditions, we treated the intestinal neuronal cell line with sh-Nupr1 under I/R conditions and assessed various parameters. We found that compared to the model + sh-NC and model + sh-Nupr1 groups, the expression of Nupr1 in intestinal neuronal cells was significantly reduced (Figure S2A). The fluorescence intensity of P62 decreased significantly, while the fluorescence intensity of LC3B increased markedly (*P* < 0.01) (Figure S2B). Moreover, the protein expression levels of Nupr1 and P62 were significantly decreased, and the ratio of LC3II/LC3I was significantly increased (*P* < 0.01) (Figure S2C). Early apoptosis rate in intestinal neuronal cells decreased significantly (*P* < 0.01) (Figure S2D). Mitochondrial membrane potential (MMP) in intestinal neuronal cells significantly increased (*P* < 0.01) (Figure S2E), along with a notable improvement in mitochondrial injury status (Figure S2F). Additionally, ROS levels decreased (*P* < 0.01) (Figure S2G). These results indicate that silencing Nupr1 can increase autophagic activity in intestinal neuronal cells and improve mitochondrial homeostasis.

In summary, these results indicate that Dex exerts a protective effect on Intestinal neuron cells during intestinal I/R injury by downregulating Nupr1 to regulate autophagy activity and improve mitochondrial function.

### Dex regulates autophagy activity and mitochondrial homeostasis via Nupr1 to alleviate intestinal I/R Injury

Eighty SD rats were divided into six groups for experimentation: control group, I/R model group, I/R + Dex1, I/R + Dex2 treatment groups, and I/R + Dex + oe NC and I/R + Dex + oe Nupr1 groups, I/R+sh-NC and I/R+sh-Nupr1. The I/R group underwent 1 h of SMA occlusion followed by reperfusion to simulate intestinal I/R injury. In the I/R + Dex1 group, Dex was administered at a rate of 2.5 µg/kg/h via tail vein infusion 1 h before I/R induction. Rats in the I/R + Dex2 group received Dex at a rate of 5.0 µg/kg/h via tail vein infusion 1 h before I/R induction. The I/R + Dex + oe NC and I/R + Dex + oe Nupr1 groups received lentivirus injections following the protocol of the Dex2 group with control lentivirus and Nupr1 expression lentivirus, respectively.

**Fig. 5:**
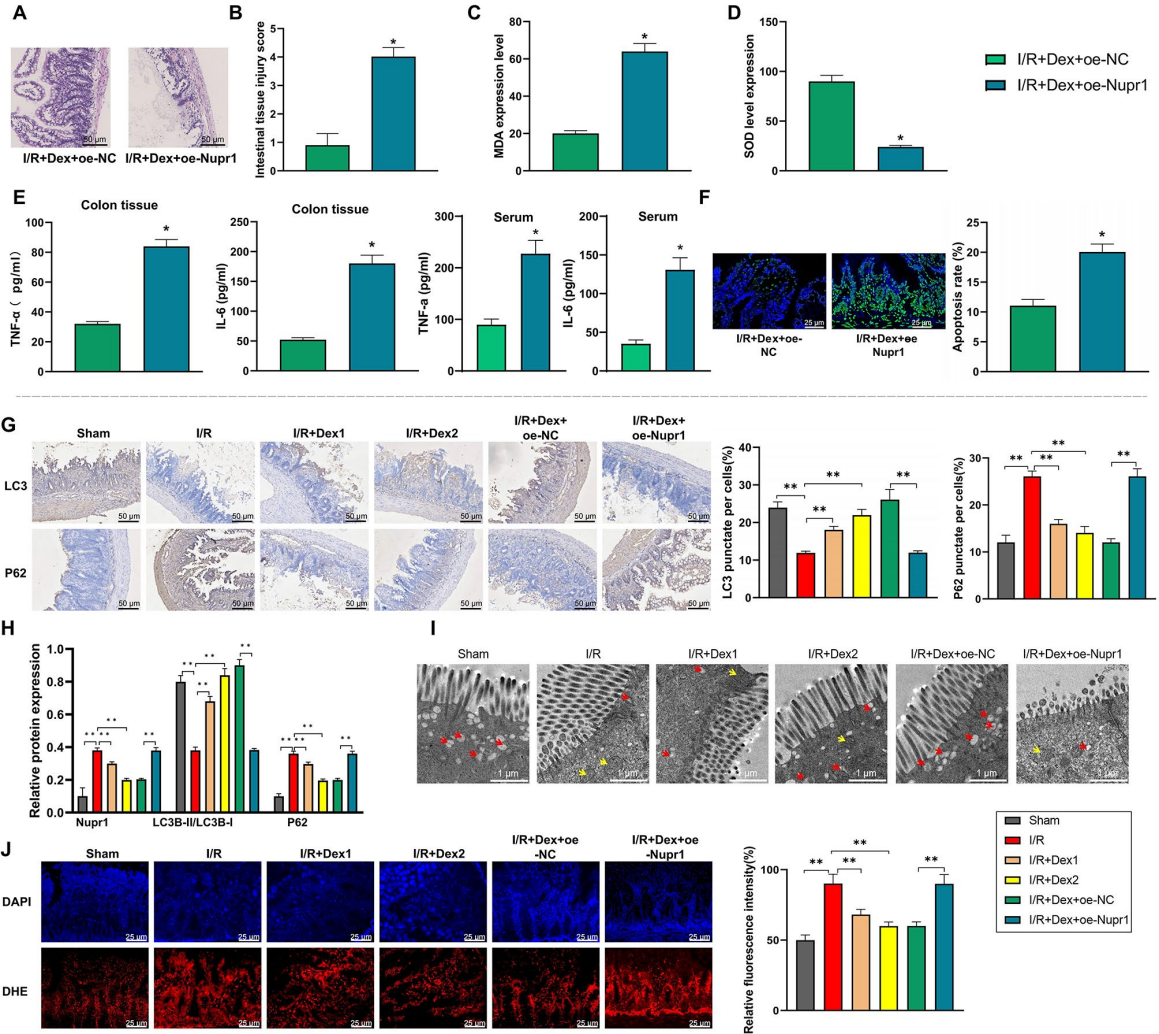
The role of Dex in the rat model of intestinal I/R injury. Note: (**A**) Observation of H&E staining results revealed differences in the extent of intestinal tissue damage between the I/R + Dex + oe NC model group rats and the I/R + Dex + oe Nupr1 Dex treatment group; (**B**) Evaluation of tissue pathological damage score (Chiu’s score) showed differences in the extent of intestinal tissue damage between the I/R + Dex + oe NC model group rats and the I/R + Dex + oe Nupr1 Dex treatment group; (**C**-**D**) Comparison of oxidative stress levels in intestinal tissues of rats in each group after Dex pretreatment; (**E**) ELISA experiment results demonstrated differences in TNF-α and IL-6 levels in the serum and colon tissues of rats between the I/R + Dex + oe NC model group and the I/R + Dex + oe Nupr1 Dex treatment group; (**F**) TUNEL staining results compared the amount of cell apoptosis in intestinal tissues of rats between the I/R + Dex + oe NC model group and the I/R + Dex + oe Nupr1 Dex treatment group; (**G**) Immunohistochemical detection of the proportion of positive cells for LC3B and p62 in sham, I/R, I/R + Dex1, I/R + Dex2, I/R + Dex + oe NC, and I/R + Dex + oe Nupr1 groups; (**H**) Western Blot analysis of Nupr1, LC3, and P62 protein expression levels in the rat intestinal in the sham, I/R, I/R + Dex1, I/R + Dex2, I/R + Dex + oe NC, and I/R + Dex + oe Nupr1 groups; (**I**) Observation of ultrastructural changes in intestinal tissues of each group using transmission electron microscopy (yellow arrows indicate mitochondria, red arrows indicate mitochondria autophagic bodies); (**J**) DHE probe staining to detect ROS levels. *P < 0.05, **P < 0.01. Cell experiments were repeated three times

Intestinal tissue samples were observed through H&E staining, whereby cell nuclei were stained blue-purple and cytoplasm pink. Microscopically, the I/R + Dex + oe-NC group displayed mild mucosal villi edema and shedding, epithelial cell exfoliation, damaged glands, and slight infiltration of neutrophils in the lamina propria. In contrast, the I/R + Dex + oe-Nupr1 group exhibited pronounced villi edema and shedding, severe glandular damage, lamina propria disintegration, significant neutrophil infiltration, and occasional bleeding (Fig. 5A). Compared to the I/R + Dex + oe-NC group, the I/R + Dex + oe-Nupr1 group showed a significant increase in intestinal tissue pathology score (Chiu’s score) (*P* < 0.05) (Fig. 5B). These results indicate that Nupr1 overexpression can reverse the protective effect of Dex against intestinal I/R injury.

By measuring the MDA concentration and total SOD activity in the intestinal mucosal tissues of each group, the oxidative stress levels in the tissues were assessed. The results indicated that compared to the I/R + Dex + oe-NC group, the MDA level in the intestinal tissues of rats in the I/R + Dex + oe-Nupr1 group was significantly elevated (*P* < 0.05). Similarly, the SOD activity level in the intestinal tissues of rats in the I/R + Dex + oe-Nupr1 group was significantly decreased compared to the I/R + Dex + oe-NC group (*P* < 0.05) (Fig. 5C-D). The ELISA results of serum and colon tissue showed that compared to the I/R + Dex + oe-NC group, the levels of TNF-α in serum and colon tissue of rats in the I/R + Dex + oe-Nupr1 group were significantly elevated (*P* < 0.05). Furthermore, the levels of IL-6 in serum and colon tissue of rats in the I/R + Dex + oe-Nupr1 group were also significantly increased compared to the I/R + Dex + oe-NC group (*P* < 0.05) (Fig. 5E).

TUNEL assay, a common method for detecting cell apoptosis, was utilized to assess the extent of cell apoptosis in the intestinal tissues. The results showed that compared to the I/R + Dex + oe-NC group, the cell apoptosis rate in the intestinal tissues of rats in the I/R + Dex + oe-Nupr1 group was significantly higher (*P* < 0.05) (Fig. 5F).

Additionally, immunohistochemical staining was employed to evaluate the expression levels of LC3B and p62-related proteins in the cell models of intestinal tissues from each group of rats. The findings demonstrated that compared to the sham group, the I/R model group exhibited a significant increase in the proportion of p62-positive cells and a notable decrease in the proportion of LC3B-positive cells (*P* < 0.01). Following treatment with Dex1 and Dex2, there was a significant decrease in the proportion of p62-positive cells and a significant increase in the proportion of LC3B-positive cells compared to the I/R model group (*P* < 0.01). Moreover, in the I/R + Dex group, overexpression of Nupr1 led to a significant increase in the proportion of p62-positive cells and a significant decrease in the proportion of LC3B-positive cells (*P* < 0.01) (Fig. 5G).

Furthermore, Western blot analysis was conducted to measure the expression levels of Nupr1 and autophagy-related proteins in the cell models of intestinal tissues from each group of rats. The results revealed that compared to the sham group, the I/R model group showed a significant increase in the expression levels of Nupr1 and P62 proteins, while the expression of LC3II/LC3I was significantly reduced (*P* < 0.01). Following treatment with Dex1 and Dex2, there was a significant decrease in the expression levels of Nupr1 and P62 proteins and a significant increase in the expression of LC3II/LC3I compared to the model group (*P* < 0.01). Additionally, in the I/R + Dex group, overexpression of Nupr1 resulted in a significant increase in the expression levels of Nupr1 and P62 proteins and a significant decrease in the expression of LC3II/LC3I (*P* < 0.01) (Fig. 5H).

Transmission electron microscopy results showed that in the sham group, intestinal tissue cells exhibited a small number of damaged mitochondria, with cristae structure disrupted or even absent. In the I/R model group, mitochondria displayed significant morphological abnormalities, including marked swelling, incomplete mitochondrial membranes, mitochondrial dissolution, and balloon-like degeneration, accompanied by a decrease in autophagosomes. Treatment with Dex1 and Dex2 improved mitochondrial conditions and increased the number of autophagosomes. However, in the I/R + Dex group, overexpression of Nupr1 led to abnormal mitochondrial morphology and a reduction in autophagosomes (Fig. 5I) (Zhang et al. 2021). DHE probe staining results demonstrated that compared to the control group, the ROS levels were elevated in the I/R model group. Following treatment with Dex1 and Dex2, the ROS levels were decreased, while in the I/R + Dex group with Nupr1 overexpression, the ROS levels were increased (Fig. 5J).

To investigate the impact of Nupr1 silencing on intestinal ischemia-reperfusion injury in rats, we used sh-Nupr1 treatment on these animals and assessed various indicators. Our findings revealed significant mucosal villi edema and shedding, severe damage to the glands, breakdown of the lamina propria, extensive infiltration of neutrophils, and in some cases, bleeding in the I/R + sh-NC group. In contrast, the I/R + sh-Nupr1 group showed milder mucosal villi edema and shedding, epithelial cell exfoliation, damaged glands, and a lower level of neutrophil infiltration in the lamina propria (Figure S3A). Compared to the I/R + sh-NC group, the rats in the I/R + sh-Nupr1 group exhibited a significantly reduced pathological injury score in the intestinal tissue (Chiu’s score) (*P* < 0.05) (Figure S3B). Additionally, the I/R + sh-Nupr1 group demonstrated a significantly lower level of MDA and a higher level of SOD activity in the intestinal tissue when compared to the I/R + sh-NC group (*P* < 0.05) (Fig. S3C and S3D). Furthermore, the levels of TNF-a and IL-6 in the serum and colonic tissue of rats in the I/R + sh-Nupr1 group were significantly lower than those in the I/R + sh-NC group (*P* < 0.05) (Figure S3E). The apoptotic rate in the intestinal tissue of rats in the I/R + sh-Nupr1 group was significantly lower compared to that in the I/R + sh-NC group (*P* < 0.05) (Figure S3F). Moreover, the proportion of P62-positive cells in the intestinal tissue of rats in the I/R + sh-Nupr1 group was significantly reduced, while the proportion of LC3B-positive cells was significantly increased compared to the I/R + sh-NC group (*P* < 0.05) (Figure S3G). The expression levels of Nupr1 and P62 proteins in the intestinal tissue of rats in the I/R + sh-Nupr1 group were significantly lower, whereas the ratio of LC3II/LC3I expression was significantly higher compared to the I/R + sh-NC group (*P* < 0.05) (Figure S3H). Treatment with sh-Nupr1 was found to improve mitochondrial status (Figure S3I) and reduce ROS levels (Figure S3J). These results suggest that Nupr1 silencing can increase autophagic activity, enhance mitochondrial homeostasis, and thereby alleviate intestinal ischemia-reperfusion injury.

These findings suggest that Dex can effectively alleviate the inflammatory response, cell apoptosis, and autophagy impairment induced by intestinal I/R injury by mediating the expression of Nupr1. Furthermore, these results support its potential therapeutic value in the treatment of intestinal I/R injury.

## Discussion

The potential therapeutic role of Dex in intestinal I/R injury has been highlighted due to its significant protective effects (Li et al. 2022a, b). Dex is a highly selective α2-adrenergic receptor agonist that exhibits anxiolytic, stabilizing hemodynamics, reducing stress responses, analgesic, antishivering, and diuretic effects (Weerink et al. 2017). Dex may prevent acute kidney injury by inhibiting mitochondrial damage and cellular inflammation (Song et al. 2021). Additionally, Dex has been shown to have a supportive role in protecting against I/R injury. Numerous studies indicate that Nupr1 plays a crucial role in autophagic activity and mitochondrial homeostasis (Fan et al. 2022; Xiao et al. 2022; Huang et al. 2021). Previous research suggests that Nupr1 can modulate dopaminergic neuron autophagy (Xu et al. 2017). Through experimental results and analysis in this study, it is evident that Dex modulates Nupr1 expression, impacting intestinal neuron autophagy and mitochondrial function, thereby ameliorating I/R-induced intestinal damage. This regulatory mechanism on autophagy and mitochondrial homeostasis offers novel insights into intestinal protection and presents new research avenues for related diseases (Hu et al. 2022a, b; Zhao et al. 2023; He et al. 2023).

Traditional studies have mainly focused on the pathophysiological changes and molecular regulatory mechanisms of inflammation response in intestinal I/R injury, with limited emphasis on the roles of autophagy and mitochondrial homeostasis (Song et al. 2024; Zhang et al. 2022). Therefore, this study delves into the protective mechanisms of intestinal I/R injury by investigating the regulatory effects of Dex on autophagy and mitochondrial function using high-throughput RNA sequencing and scRNA-seq technologies (Hou et al. 2023; Yang et al. 2021).

Autophagy is a tightly regulated intracellular degradation process that handles damaged organelles and protein misfolding through the sequestration of autophagosomes followed by lysosomal degradation. It plays a crucial role in maintaining overall homeostasis by modulating inflammation, oxidative stress, and cell apoptosis in various diseases, including ischemia-reperfusion injury (Wen et al. 2019). In certain ischemic conditions, reduced autophagy typically enhances the production of pro-inflammatory cytokines, promotes the accumulation of damaged cellular components, and leads to excessive inflammatory responses, oxidative stress, and cell apoptosis (Murrow and Debnath 2013). Moreover, autophagic activation can significantly ameliorate ischemic injuries by directly processing dysfunctional intracellular components and aggregated inflammasomes, thereby reducing cell death and inflammation. Therefore, appropriate activation of autophagy may be a feasible therapeutic strategy for ischemia-reperfusion. Generally, autophagy as a feedback mechanism is activated in the presence of oxidative stress and participates in the clearance of reactive oxygen species (ROS) by degrading dysfunctional mitochondria. Autophagy also regulates cell apoptosis by degrading anti-apoptotic factors or damaged molecules and organelles in mammalian cells (Yang et al. 2018). Dysfunctional autophagy is closely associated with intestinal mucosal barrier damage, antimicrobial peptide secretion disorders, and intestinal inflammation, while autophagic activation exerts protective effects against ischemia-reperfusion-induced injuries by inhibiting inflammation, oxidative stress, and cell apoptosis (Wu et al. 2019).

Previous studies have shown that autophagy is suppressed in intestinal ischemia-reperfusion injuries, and esculetin can protect against inflammation, oxidative stress, and cell apoptosis in intestinal I/R injuries by activating the SIRT3/AMPK/mTOR signaling pathway and autophagy (Shen et al. 2023). Intestinal I/R injury post ischemia is related to the activation of the JAK2/STAT3 pathway and beclin-1-mediated autophagy inactivation, and AG490 inhibiting the JAK2/STAT3 pathway reactivates autophagy, enhancing survival after intestinal ischemia-reperfusion injury (Liu et al. 2022a, b). Promoting autophagy may enhance intestinal epithelial barrier function after intestinal ischemia-reperfusion by inhibiting claudin-2 expression and promoting occludin expression (Li et al. 2022a, b). Sometimes, autophagy may also exhibit negative effects, such as promoting cell apoptosis or inducing inflammatory responses. Studies have indicated that after intestinal ischemia-reperfusion, the intestinal AMPK-Sirt1 autophagy pathway is activated, and propofol further activates this pathway, alleviating intestinal injury, suppressing cell apoptosis, reversing inflammation and oxidative stress, improving the 24-hour survival rate of rats post intestinal ischemia-reperfusion, and mitigating intestinal injuries (Liu et al. 2022a, b). The dual role of autophagy in intestinal ischemia-reperfusion may depend on factors such as the timing, intensity, and regulatory mechanisms of autophagic activation.

Prior research has paid scant attention to the role of neurons in intestinal damage and the regulation of neuron autophagy and mitochondrial function. By significantly upregulating LC3-II protein expression and decreasing P62 and Nupr1 protein expression, this study validates Dex’s regulatory effects on neuron autophagy and mitochondrial homeostasis, providing new evidence for the importance of neurons in intestinal damage and expanding the understanding of intestinal protection mechanisms.

Additionally, in vivo experiments in rats revealed that Dex treatment resulted in milder intestinal tissue damage, significantly reduced cell apoptosis, downregulation of inflammatory factor expression levels, and decreased oxidative stress levels. These series of experiments confirm the protective effects of Dex against intestinal I/R injury, laying a solid experimental foundation for clinical treatment (Hou et al. 2023; Yang et al. 2021; Garcia-Alonso et al. 2023).

In contrast to previous studies, this research underscores Dex’s capability to enhance neuron autophagy activity and improve mitochondrial homeostasis by downregulating Nupr1, thereby safeguarding the intestine from I/R-related damage. This study emphasizes the significance of neurons in intestinal damage and presents a new mechanistic explanation for intestinal protection, diverging from the conventional inflammation-focused studies, and providing new insights (Moody et al. 2021; Hashiya et al. 2023; Sun et al. 2023).

**Fig. 6:**
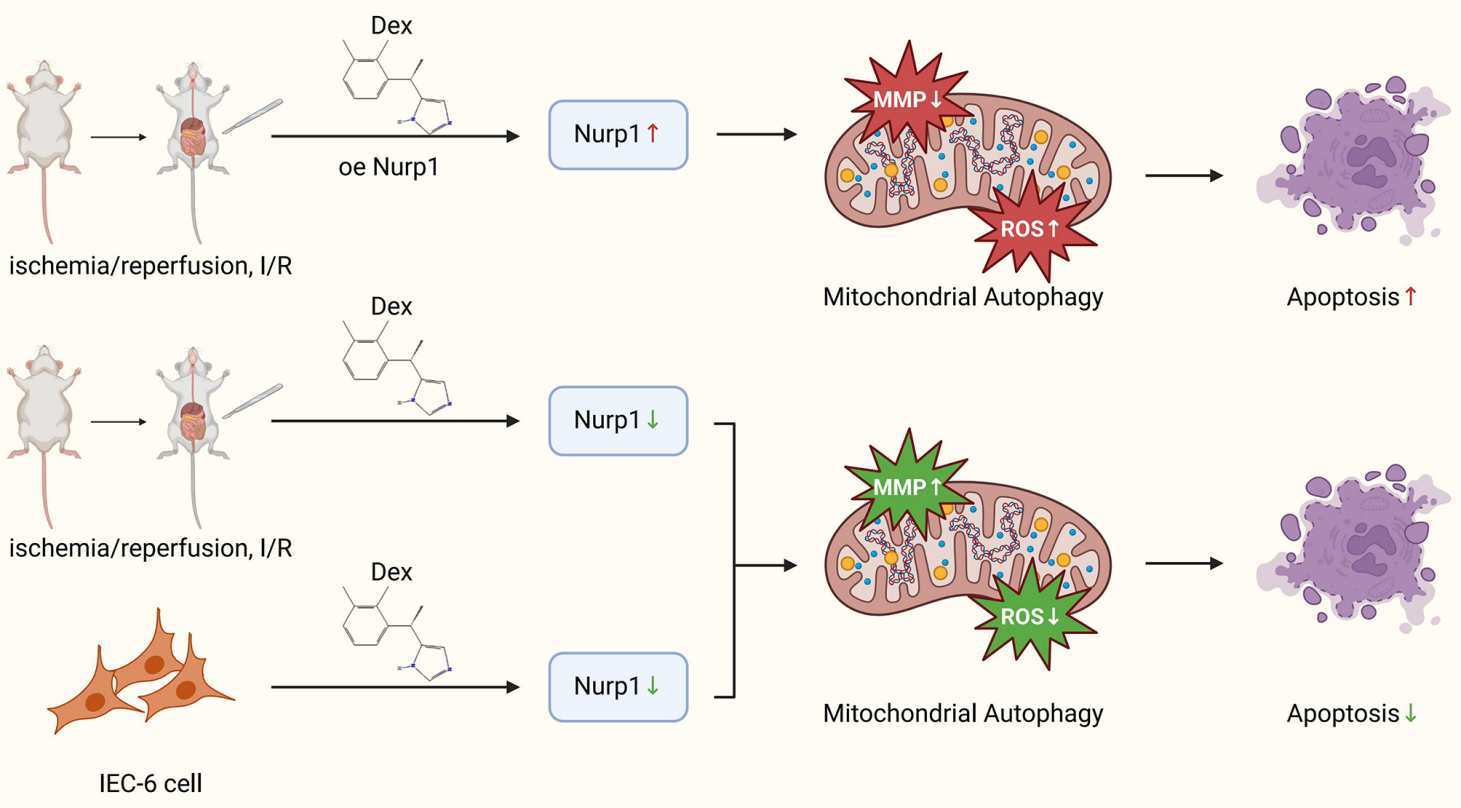
Dex regulates intestinal neuron autophagy activity and mitochondrial homeostasis via Nupr1 to alleviate intestine I/R injury

This study utilized multi-omics analysis to elucidate the protective mechanisms of Dex in intestinal I/R injury, particularly focusing on its role in modulating intestinal neuron autophagy and mitochondrial homeostasis (Fig. 6). It was discovered that Dex downregulates Nupr1, enhances intestinal neuron autophagy activity, and improves mitochondrial function, thereby alleviating intestinal damage caused by I/R. In vitro experiments demonstrated that Dex treatment significantly increased the expression of autophagy-related proteins and improved mitochondrial membrane potential while reducing ROS levels. In vivo experiments on rats treated with Dex exhibited lower intestinal tissue damage, reduced cell apoptosis, and downregulation of inflammatory factors. These findings provide new molecular insights into the clinical treatment of intestine I/R injury with Dex.

The scientific and clinical significance of this study lies in the in-depth exploration of Dex’s protective mechanisms against intestinal I/R injury, particularly through its role in modulating intestinal neuron autophagy activity and mitochondrial homeostasis to mitigate damage. Using techniques such as high-throughput RNA sequencing and scRNA-seq, the research revealed that Dex, by downregulating Nupr1, markedly improves intestinal tissue damage, reduces cell apoptosis, and lowers oxidative stress levels, offering new molecular mechanisms for treating intestine I/R injury. These findings not only establish a foundation for further research on therapeutic mechanisms of intestinal damage but also provide crucial insights for the development of related drugs and treatment strategies.

However, this study has limitations, such as findings only being validated in animal models and in vitro cell experiments, requiring further related research to confirm their effectiveness and safety in clinical applications. Additionally, although the study confirmed Dex’s protective effect against intestinal damage, the specific therapeutic mechanisms still necessitate deeper investigations in animal models and clinical trials for validation.

Looking ahead, future research could delve into the long-term effects of Dex on intestinal damage and its potential applications in different types of intestinal diseases. Furthermore, exploring more effective combination therapy approaches by integrating Dex with other drugs or treatment modalities could enhance the comprehensive treatment outcomes for intestine I/R injury. Moreover, further research on the role of Nupr1 in intestinal diseases could offer more possibilities for developing targeted treatment strategies.

## Electronic supplementary material

Below is the link to the electronic supplementary material.

Supplementary Material 1: Figure S1 Quality control, variance analysis, and PCA of scRNA-seq data. Note: (A) Quality control of cells from the intestinal tissue samples of three normal rats, with scatterplots for the number of genes (nFeature_RNA), RNA molecules (nCount_RNA), and percentage of mitochondrial genes (percent.mt) for each cell; (B) Correlation between nCount and percent.mt in cells (left) and between nCount and nFeature (right); (C) Variance analysis to identify highly variable genes in cells, with red dots representing highly variable genes and black dots representing invariant genes; (D) Cluster analysis of cells from each sample based on principal component analysis; (E) Heat map showing the expression of genes comprising the top 2 principal components; (F) Scatter plot showing genes composing the top 2 principal components; (G) Comparison of p-values for each principal component using the JackStrawPlot function; (H) Determination of principal components for subsequent analysis using the ElbowPlot function, identifying the inflection point based on variance changes, with more important principal components having larger standard deviations

Supplementary Material 2: Fig. S2 Effects of Nupr1 Silencing on Autophagic Activity and Mitochondrial Function in Intestinal Neuronal Cell Lines. Note: (A) Quantitative PCR measurement of nupr1 expression in the cellular model; (B) Immunofluorescence analysis of LC3B and p62 protein levels in various groups of intestinal neuronal cells; (C) Western Blot analysis of Nupr1, LC3, and p62 protein expression levels in different groups of intestinal neuronal cells; (D) Flow cytometry observation of early apoptosis rate in each group of cells; (E) Hoechst 33,342 staining for apoptosis evaluation in cells of each group; (F) Mitochondrial membrane potential using mmp detection; (G) Transmission electron microscopy observation of ultrastructure changes in cells of each group, with yellow arrows indicating mitochondria and red arrows indicating autophagosomes; (H) H2DCFDA staining for ros level detection. **P < 0.01. The cell experiments were repeated three times

Supplementary Material 3: Fig. S3. The Role of Nupr1 Silencing in the Rat Model of I/R. Note: (A) H&E staining for assessment of intestinal tissue damage in rat groups; (B) Utilization of tissue pathology damage score (chiu’s score) for evaluation of severity of intestinal tissue damage in rats; (C-D) Oxidative stress levels in intestinal tissues of various rat groups; (E) Elisa experiment for detection of discrepancies in tnf-α and il-6 levels in rat serum; (F) TUNEL staining for evaluation of apoptotic cell numbers in intestinal tissues of respective rat groups; (G) Immunohistochemistry for determination of proportion of positive cells for lc3b and p62 in intestinal tissues of each rat group; (H) Western blot analysis for quantification of expression levels of Nupr1, LC3, and p62 proteins in intestinal tissues of each rat group; (I) Transmission Electron Microscopy for observation of changes in ultrastructure in intestinal tissues of all groups (Yellow arrows indicate mitochondria, red arrows indicate autophagosomes); (J) DHE probe staining for measurement of ROS Levels. *P < 0.05, **P < 0.01. Cell experiments were repeated three times

Supplementary Material 4

## Data Availability

The datasets used or analyzed during the current study are available from the corresponding author on reasonable request.
