# Supplementary figures and images for: Dexmedetomidine alleviates intestinal ischemia/reperfusion injury by modulating intestinal neuron autophagy and mitochondrial homeostasis via Nupr1 regulation
Source: Mol Med. 2024 Nov 6;30:203. doi: 10.1186/s10020-024-00952-2 (PMC11542338; doi:10.1186/s10020-024-00952-2)

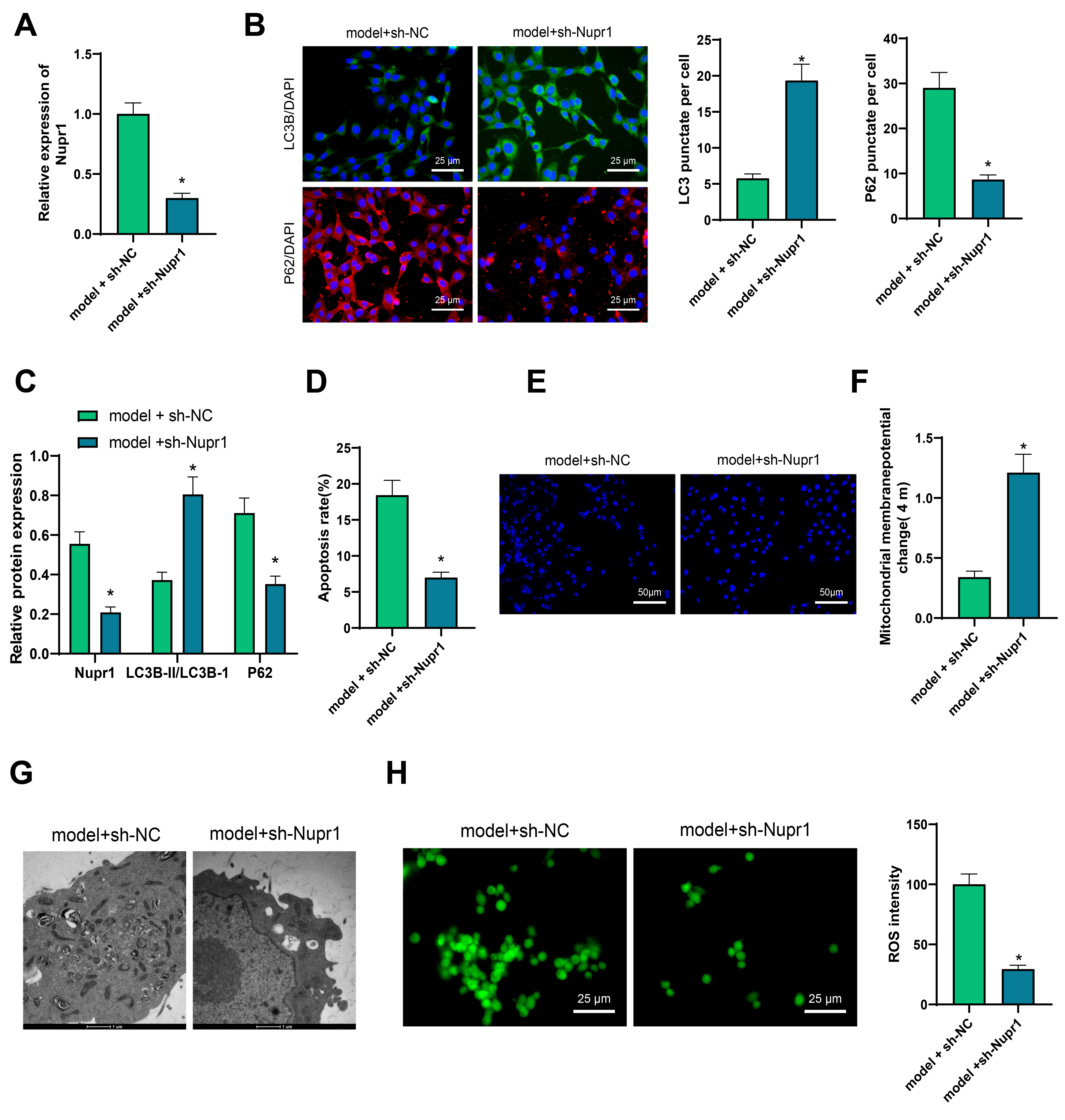

Supplement: Supplementary file 2 — Supplementary Material 2: Fig. S2 Effects of Nupr1 Silencing on Autophagic Activity and Mitochondrial Function in Intestinal Neuronal Cell Lines. Note: (A) Quantitative PCR measurement of nupr1 expression in the cellular model; (B) Immunofluorescence analysis of LC3B and p62 protein levels in various groups of intestinal neuronal cells; (C) Western Blot analysis of Nupr1, LC3, and p62 protein expression levels in different groups of intestinal neuronal cells; (D) Flow cytometry observation of early apoptosis rate in each group of cells; (E) Hoechst 33,342 staining for apoptosis evaluation in cells of each group; (F) Mitochondrial membrane potential using mmp detection; (G) Transmission electron microscopy observation of ultrastructure changes in cells of each group, with yellow arrows indicating mitochondria and red arrows indicating autophagosomes; (H) H2DCFDA staining for ros level detection. **P < 0.01. The cell experiments were repeated three times [file 10020_2024_952_MOESM2_ESM.jpg]

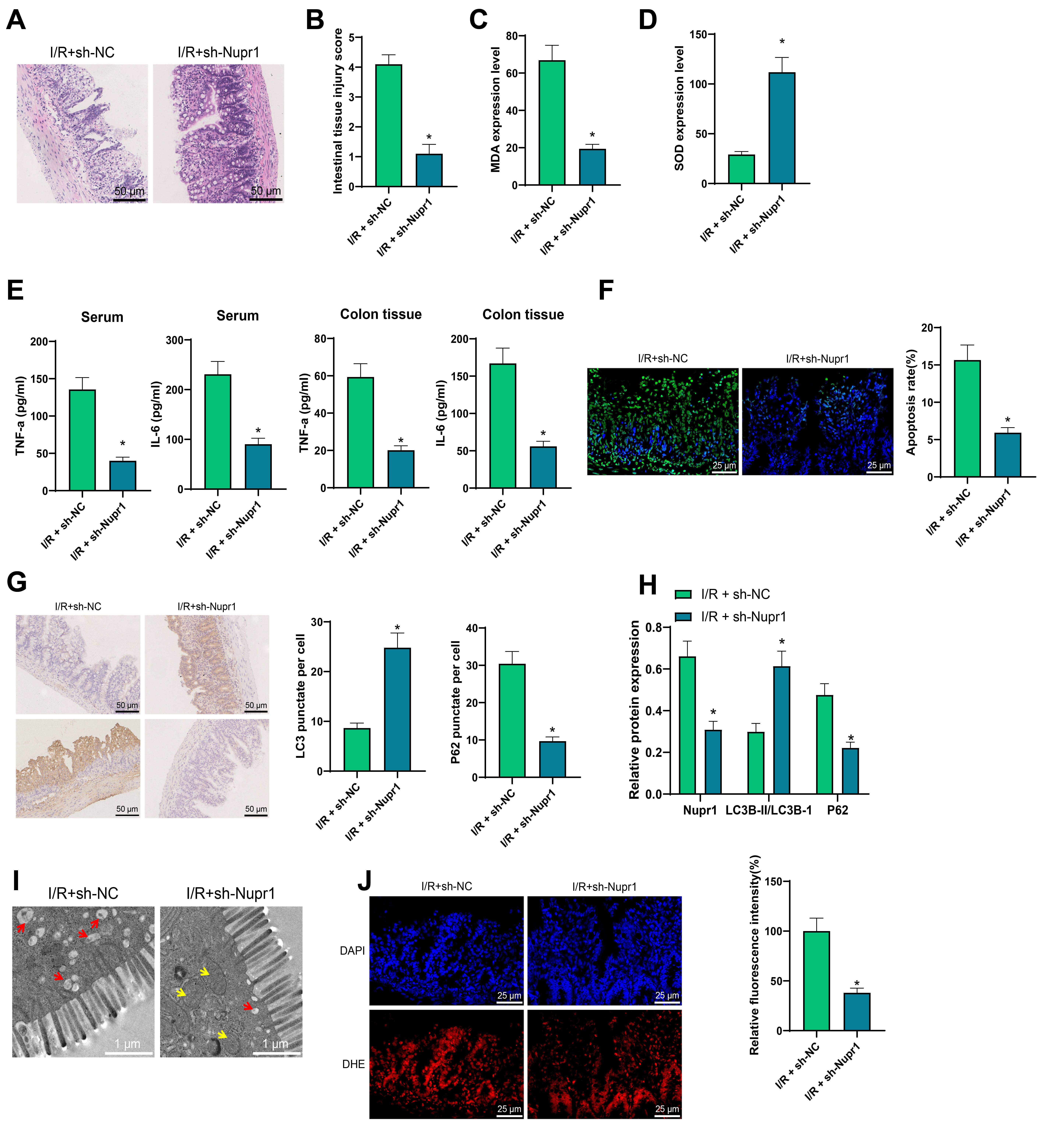

Supplement: Supplementary file 3 — Supplementary Material 3: Fig. S3. The Role of Nupr1 Silencing in the Rat Model of I/R. Note: (A) H&E staining for assessment of intestinal tissue damage in rat groups; (B) Utilization of tissue pathology damage score (chiu’s score) for evaluation of severity of intestinal tissue damage in rats; (C-D) Oxidative stress levels in intestinal tissues of various rat groups; (E) Elisa experiment for detection of discrepancies in tnf-α and il-6 levels in rat serum; (F) TUNEL staining for evaluation of apoptotic cell numbers in intestinal tissues of respective rat groups; (G) Immunohistochemistry for determination of proportion of positive cells for lc3b and p62 in intestinal tissues of each rat group; (H) Western blot analysis for quantification of expression levels of Nupr1, LC3, and p62 proteins in intestinal tissues of each rat group; (I) Transmission Electron Microscopy for observation of changes in ultrastructure in intestinal tissues of all groups (Yellow arrows indicate mitochondria, red arrows indicate autophagosomes); (J) DHE probe staining for measurement of ROS Levels. *P < 0.05, **P < 0.01. Cell experiments were repeated three times [file 10020_2024_952_MOESM3_ESM.jpg]
