# Supplementary material for: Dexmedetomidine alleviates intestinal ischemia/reperfusion injury by modulating intestinal neuron autophagy and mitochondrial homeostasis via Nupr1 regulation
Source: Mol Med. 2024 Nov 6;30:203. doi: 10.1186/s10020-024-00952-2 (PMC11542338; doi:10.1186/s10020-024-00952-2)
Supplement: Supplementary file 4 — Supplementary Material 4 [file 10020_2024_952_MOESM4_ESM.docx]

Table S1. RT-qPCR primer sequence

| Gene | Sequence(5’-3’) |
| --- | --- |
| Nupr1(rat) | Forward: AGGACCTAGGCCTGCTTGAT |
|  | Reverse: TTCTCGCTCCTAACTTGCCC |
| GAPDH(rat) | Forward: GGACCTCATGGCCTACATGG |
|  | Reverse: ATTCGAGAGAAGGGAGGGCT |
